# A critical role for cortical amygdala circuitry in shaping social encounters

**DOI:** 10.21203/rs.3.rs-3015820/v1

**Published:** 2023-07-06

**Authors:** Russo Scott, Antonio Aubry, Romain Durand-de Cuttoli, Fisher-Foye Rachel, Parise Lyonna, Flurin Cathomas, C Burnett, Yewon Yang, chongzhen yuan, Alexa Lablanca, Kenny Chan, Hsiao-yun Lin, Robert Froemke, Long Li

**Affiliations:** Icahn School of Medicine at Mount Sinai; Icahn School of Medicine at Mount Sinai; Icahn School of Medicine at Mt. Sinai; University of Palermo; Icahn School of Medicine at Mount Sinai; Icahn School of Medicine at Mount Sinai; Icahn School of Medicine at Mt. Sinai; Icahn School of Medicine at Mt. Sinai; Icahn School of Medicine at Mt. Sinai; Icahn School of Medicine at Mount Sinai; Icahn School of Medicine at Mount Sinai; NYU; Icahn School of Medicine at Mount Sinai

## Abstract

Aggression is an evolutionarily conserved behavior that controls social hierarchies and protects valuable resources like mates, food, and territory. In mice, aggressive behaviour can be broken down into an appetitive phase, which involves approach and investigation, and a consummatory phase, which involves biting, kicking, and wrestling. By performing an unsupervised weighted correlation network analysis on whole-brain c-Fos expression, we identified a cluster of brain regions including hypothalamic and amygdalar sub-regions and olfactory cortical regions highly co-activated in male, but not female aggressors (AGG). The posterolateral cortical amygdala (COApl), an extended olfactory structure, was found to be a hub region based on the number and strength of correlations with other regions in the cluster. Our data further show that estrogen receptor 1 (ESR1)-expressing cells in the COApl exhibit increased activity during attack behaviour, and during bouts of investigation which precede an attack, in male mice only. Chemogenetic or optogenetic inhibition of COApl ESR1 cells in AGG males reduces aggression and increases pro-social investigation without affecting social reward/reinforcement behavior. We further confirmed that COApl ESR1 projections to the ventrolateral portion of the ventromedial hypothalamus and central amygdala are necessary for these behaviours. Collectively, these data suggest that in aggressive males, COApl ESR1 cells respond specifically to social stimuli, thereby enhancing their salience and promoting attack behaviour.

Aggressive behaviours are complex forms of social behaviour that have many functions, including acquiring resources to help ensure survival. These behaviours are composed of an appetitive and consummatory phase^1^. In mice, the appetitive phase involves approaching and investigating the conspecific, while the consummatory phase involves a series of motor behaviours such as biting, kicking or wrestling^2,3^. Our lab has previously characterized a population of outbred mice that exhibit individual differences in aggressive vs. pro-social behaviour in the resident-intruder (RI) test,allowing us to evaluate the underlying neural mechanisms^3–7^. While studies have identified several brain regions within the diencephalon and ventral striatum which enable aggressive behaviour^4,6,8–10^, little work has been done to identify the brain regions that play a role in the transition between appetitive and consummatory phases of aggressive behaviours. Understanding the mechanisms of this transition is vital, as it is one of the processes which distinguishes aggressive mice (AGGs) from those that do not fight (NONs). Sensory processing has been shown to be critical in the transition between these phases during other social behaviours such as parenting and social recognition^11,12^. However, whether or not activity in sensory processing cortical regions drives differences in aggressive social behaviour is unknown.

We first utilized an iDISCO+ clearing procedure followed by whole-brain immunostaining to identity c-Fos^+^ cells. We then performed an unsupervised whole-brain computational approach^13,14^ that identified a cluster of hypothalamic, amygdalar, and olfactory cortical regions highly co-activated in male, but not female AGGs. Within this cluster, we found that the posterolateral cortical amygdala (COApl), which is part of the olfactory cortex, is a key node or “hub region” that is highly connected within the broader network of this cluster. We further investigated the mechanisms by which the COApl mediates aggressive social behavior using a variety of circuit techniques. We found that COApl ESR1^+^ neurons are highly active during bouts of social investigation which precede an attack, and that this region is necessary to make the transition from the appetitive phase (investigation) of aggressive interactions to the consummatory phase (attack).

## The COApl is a key node in a male AGG network

**Figure 1:**
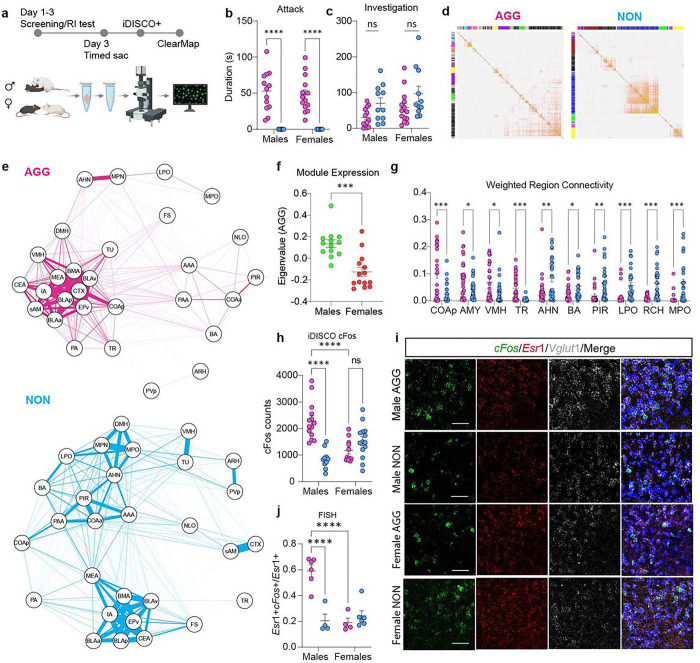
The COApl is a key node in a differentially connected module in aggressive males. **a**, Experimental timeline. **b**, Aggression duration in male and female AGGs were significantly higher than in NONs (main effect of phenotype, $F_{\left(1,45\right)}=88.31$, p<0.0001). **c**, NONs of both sexes exhibited more social investigation than AGGs (main effect of phenotype, $F_{\left(1,45\right)}=7.686$, p=0.0081, all post-hoc comparisons were p>0.05) n = 13 (Male AGG), 14 (Female AGG), 11 Male NON), 11 (Female NON). **d**, Whole-brain c-Fos network analysis in AGG and NON mice. Each module is arbitrarily assigned a unique colour label in the left and top bars of the topological overlap matrix (TOM). Darker colours indicate stronger c-Fos co-expression between brain regions. **e**, Network plot of the pink module. Module preservation analysis indicated that this module was the least preserved in terms of module connectivity in the NON network relative to the AGG network. **f**, Male AGGs exhibited higher pink module expression than female AGGs, $t_{\left(25\right)}=4.612$, p=0.0001) **g**, Regions in the pink module which differed in intramodular connectivity between AGG and NON networks. Regions are ordered from highest to lowest average connectivity in the AGG network. Each point represents the weighted connectivity between the listed region and another region within the pink module. All q values were less than 0.05. **h-j**, c-Fos expression in the COAp. Both iDISCO+ (**h**) and FISH (**j**) revealed a significant phenotype × sex interaction (iDISCO+: $F_{\left(1,45\right)}=29.16$, p<0.0001; FISH: $F_{\left(1,14\right)}=28.93$, p<0.0001) with male AGGs exhibiting higher c-Fos than male NONs (iDISCO: p<0.0001. FISH: p=0.0002, Tukey’s post-hoc) and female AGGs (iDISCO+: p<0.0001. FISH: p<0.0001, Tukey’s post-hoc). Male AGGs also exhibited a higher number of c-Fos^+^ cells expressing Esr1 (phenotype x sex interaction: $F_{\left(1,12\right)}=10.56$, p=0.007) than male NONs (p=0.0067, Tukey’s post-hoc) and female AGGs (p=0.0032, Tukey’s post-hoc) n = 6 (Male AGG), 4 (Female AGG), 4 Male NON), 6 (Female NON) * p < 0.05, ** p < 0.01, *** p < 0.01, **** p < 0.001) See regions table for abbreviations.

Following a RI test, we conducted a cleared whole-brain c-Fos mapping experiment using the iDISCO+ method combined with a network analysis commonly used in RNA-Seq studies to identify differentially activated regions between male and female AGG and NON mice (Fig. 1a). Prior to brain collection, male and female AGGs and NONs were exposed to an intruder mouse. We did not see a difference in total attack duration or investigation duration between either sex (Fig. 1b, c), but we did observe that males and females engaged in distinct actions which made up the total duration metrics as previously reported^3^ (Extended Fig. 1a-f). We then assembled a single network for both AGG and NON mice (Fig. 1d). Each colour along the top and left side of the network indicates a module of highly clustered brain regions. Following the construction of each network, we performed a module preservation analysis using the AGG network as the reference network and the NON network as the test network. We found that the “pink” module was the least preserved module in the NON network (Fig. 1e; Extended Fig. 2a-c). Inspection of this module showed that sub-regions of the amygdala and hypothalamus are highly interconnected in the AGG network. In the NON network however, sub-regions of the hypothalamus and amygdala are highly connected amongst themselves, but not between the amygdala and hypothalamus. Interestingly, male AGGs expressed this module, as determined by the eigenvalues, significantly more than female AGGs (Fig. 1f). We also found that many of the top regions which showed a significant increase in c-Fos counts were different between male and female AGGs (Extended Fig. 2e, f). Of the 28 regions in the pink module, 11 regions differed in their total connectivity within the pink module between AGG and NON mice. Of the 4 regions which displayed higher connectivity in the AGG network, the COAp had the highest average connectivity (Fig. 1g). Further investigation of the COAp determined that male AGGs had more c-Fos^+^ cells in this region compared to female AGGs and male NONs in the iDISCO+ data set (Fig. 1h). Since Clearmap cannot distinguish between the lateral and medial Cortical Amygdala, we manually cropped the lateral and medial portions of the COAp and found that male AGGs showed a significant increase in the number of cells expressing cfos in the lateral relative to the medial portion of the COAp (Extended Fig. 3A). Although cFos is also up in the COApm in AGGs relative to NONs, later functional validation studies with DREADDS shown in Extended Fig (5J-I) suggest only the COApl regulates aggression. When focusing on the COApl found that male AGGs showed a significant increase in the proportion of *Esr*1 cells which expressed c-Fos relative to female AGGs and male NONs (Fig. 1i–j). Detailed analyses of this cell population determined that the majority of *Esr1*^+^ cells were glutamatergic (~ 90%) and were equally expressed in the COApl in all four groups (Extended Fig. 3a-c). We also found that the majority of cells which expressed c-Fos also expressed *Esr*1 (~ 85%; Extended Fig. 3d).

**Figure 2:**
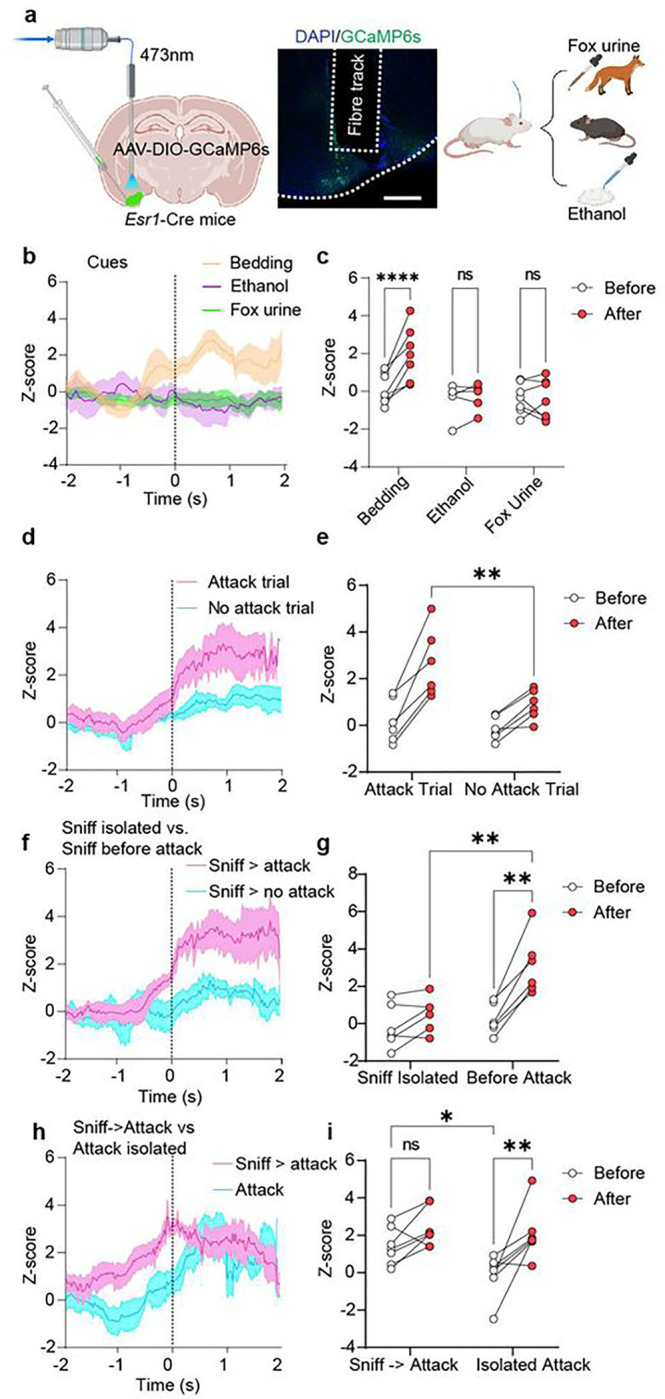
*In vivo* activity of COApl^Esr1^ cells during distinct bouts of investigation in male AGGs **a**, Experimental timeline. **b**, Peri-event plot of COApl^Esr1^ activity two seconds before and two seconds after the investigation of an odour; **c**, Quantification of activity before and after the event. There was a main effect of cue ($F_{\left(2,16\right)}=5.407$, p=0.0161) and a cue x time interaction ($F_{\left(2,16\right)}=11.91$, p=0.0007). Post-hoc comparisons revealed an increase in COApl^Esr1^ activity only during the investigation of soiled bedding (p<0.0001) **d, f, h**, peri-event plot of COApl^Esr1^ activity four seconds before and four seconds after performing the behaviour of interest; **e, g, i**, quantification of activity before and after the event. When combining all bouts of investigation on days in which an attack did not occur compared to bouts of investigation on a day in which an attack occurred, there was a significant increase in activity during investigation on attack day (main effect of trial: $F_{\left(1,5\right)}=45.56$, p=0.0010; trial × time interaction: $F_{\left(1,5\right)}=10.31$, p=0.0237, d & e) compared to investigation bouts during a no-attack day (p=0.0093, Tukey’s post-hoc test, d & e). When examining investigation bouts during attack days, it was found that there was a significant increase in COApl^Esr1^ activity during investigation bouts which preceded an attack (behaviour × time interaction: $F_{\left(1,5\right)}=25.17$, p=0.0040, f & g) compared to those which occurred in isolation (p=0.0010, Tukey’s post-hoc, f & g). When examining attack bouts, we found a significant difference in COApl^Esr1^ activity before the onset of attack when mice were engaged in investigation prior to the attack (behaviour × time interaction: $F_{\left(1,6\right)}=5.668$, p=0.0547) compared to when they were not investigating (p=0.0213, Tukey’s post-hoc, h & i). Once the mice initiated the attack, we did not see any significant difference in COApl^Esr1^ activity whether or not it was preceded by a bout of investigation (p=0.8233, Tukey’s post-hoc, h & i) n=7, * p < 0.05, ** p < 0.01, *** p < 0.001, **** p < 0.001.

To further investigate COApl^Esr1^ neuron activity *in vivo* during aggressive encounters with an intruder mouse, we injected Cre-dependent adeno-associated virus (AAV)-DIO-GCaMP6s into the COApl of *Esr1*-Cre transgenic mice to label COApl^Esr1+^ neurons (Fig. 2a). We then measured fluorescent Ca^2+^ activity by fibre photometry across three days of the RI test in male and female AGGs. Since the COApl receives input from the main olfactory bulb, we first examined the response of COApl^Esr1^ neurons to three different odours (i.e., social, non-social, and predatory scents). We found that COApl^Esr1^ neurons displayed a significant increase in Ca^2+^ activity during the investigation of soiled bedding from a cage of same-sex C57 mice but not when investigating fox urine or ethanol, which is consistent with previous literature^15^ (Fig. 2b, c). We observed similar results in female mice, with a significant increase in Ca^2+^ activity during the investigation of soiled bedding from a cage of same-sex mice (Extended Fig. 4a) but not during the investigation of fox urine or an ethanol swab (Extended Fig. 4b, c).

During the RI test, we observed that COApl^Esr1^ neurons displayed a significant increase in Ca^2+^ activity during bouts of investigation in trials where an attack occurred relative to bouts of investigation during trials in which no attack occurred (Fig. 2d, e). In contrast, we observed the opposite effect in females: COApl^Esr1^ neurons displayed a significant increase in Ca^2+^ activity during investigation trials in which no attack occured (Extended Fig. 4d). When examining individual investigation bouts during trials in which an attack occurred, we found that COApl^Esr1^ neurons increased Ca^2+^ activity only during bouts of investigation which preceded an attack compared to bouts of investigation which occurred in isolation (Fig. 2f, g). In females, we did not detect any differences in Ca^2+^ activity in female AGGs between the two types of investigation (Extended Fig. 4e). Lastly, when examining attack bouts, we found that when attacks were preceded by a bout of investigation, there was no further increase in Ca^2+^ activity during the attack. When an attack occurred spontaneously without prior investigation, we found that there was a significant increase in Ca^2+^ activity during the attack in male (Fig. 2h, i), but not in female, AGGs (Extended Fig. 4f). In a separate cohort, we recorded Ca^2+^ activity during social encounters across the four phases of the oestrous cycle but did not observe any differences in the signal during attack or investigatory behaviours as a function of cycle stage (Extended Fig. 4g-i).

## Manipulating COApl^Esr1^ neurons alters consummatory drive in male AGGs

**Figure 3:**
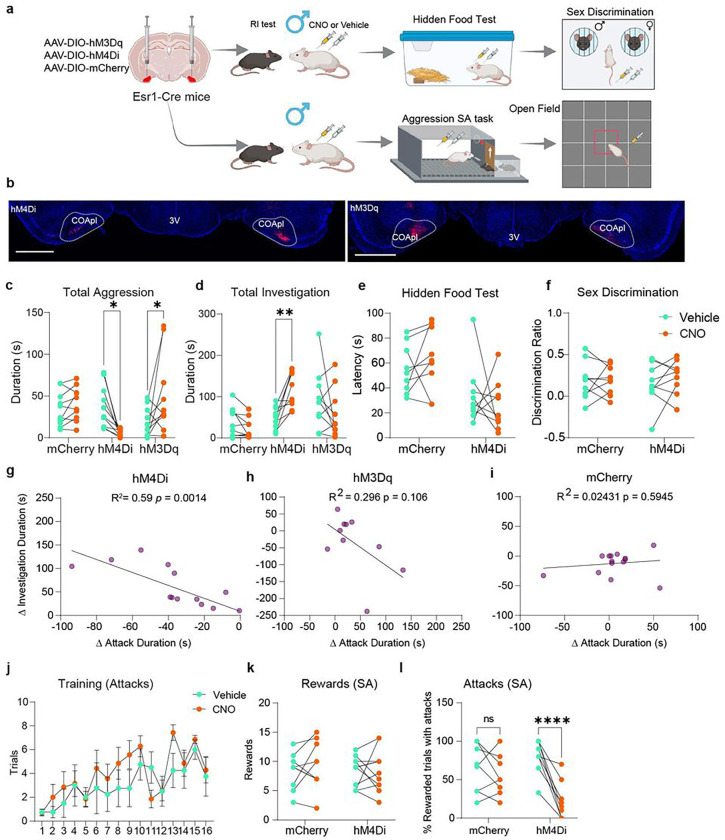
Manipulation of COApl^Esr1^ cells alters consummatory drive in male AGGs. **a**,Experimental timeline. **b**, Virus expression in COApl. **c-d**, Inhibition of COApl^Esr1^ cells significantly decreased aggression (**c**, virus × drug interaction: $F_{\left(2,27\right)}$, p=0.0007; Sidak’s post-hoc p=0.0133) and increased investigation (**d**, virus × drug interaction: $F_{\left(2,27\right)}=8.343$, p=0.0015; Sidak’s post-hoc, p=0.0035). Excitation of COApl^Esr1^ cells significantly increased aggression (Sidak’s post-hoc, p=0.014) without affecting investigation (Sidak’s post hoc, p=0.2174) n = 10 (HM4di), n = 10 (HM3dq), n = 10 (mCherry). **e-f**, Inhibition of COApl^Esr1^ cells did not affect the latency to find hidden food (**e**, virus × drug interaction: $F_{\left(1,16\right)}=1.192$, p=0.2911) or affect sex discrimination (**f**, virus × drug interaction: $F_{\left(1,16\right)}=.5122$, p=0.4845) n = 9 (HM4di), n = 9 (mCherry). **g-i**, In mice injected with hM4Di, we observed a significant negative correlation between the change in aggression and the change in investigation (**g**, r=-0.7668, $R^{2}=0.5880$, p=0.0014), but not in mice injected with hM3Dq (**h**, r=-0.5413, $R^{2}=0.2930$, p=0.1061) or mCherry (**i**, r=0.1559, $R^{2}=0.2431$, p=0.5945). **j**, Aggression self-administration experiments, both groups increased the number of rewarded trials with attacks (j, main effect of session: $F_{\left(15,135\right)}=5.412$, p<0.0001) equally. **k**, Inhibition of COApl^Esr1^ cells had no effect on lever pressing (k, virus × drug interaction: $F_{\left(1,18\right)}=0.3147$, p=0.5817). **l**, However, inhibition of COApl^Esr1^ led to a significant reduction in the percentage of rewarded trials which led to an attack (l, virus × drug interaction: $F_{\left(1,18\right)}=13.60$, p=0.0022; Sidak’s post hoc: p<0.0001) n = 11 (HM4di), n = 9 (mCherry). * p < 0.05, ** p < 0.01, *** p < 0.001, **** p < 0.001.

The results discussed above suggest that COApl^Esr1^ neurons are highly active during the transition from the appetitive phase of aggressive social interactions to the consummatory phase. To test the hypothesis that this cell population mediates this transition, we utilized viral vectors expressing designer receptors exclusively activated by designer drugs (DREADDs) to bidirectionally manipulate the activity of COApl^Esr1^ neurons. ~3–4 weeks before the RI and social self-administration (SA) tests, we injected AAV-DIO-hM3Dq, AAV-DIO-hM4Di or AAV-DIO-mCherry viruses into the COApl of 8-week-old *Esr1*-Cre mice (Fig. 3a, b). We found that inhibition of COApl^Esr1^ neurons led to a striking reduction of total attack duration and a concomitant increase in total investigation. Conversely, we found that exciting COApl^Esr1^ neurons led to a significant increase in total attack duration without a decrease in total investigation (Fig. 3c, d). We also injected AAV-DIO-hM4Di into the medial portion of the COAp and did not see any changes in attack behavior (Supplementary Fig. 5m), which is consistent with a previous lesion study in rats^16^. When examining distinct aggressive actions, we found that the number of bites during RI was affected most by the inhibition or excitation of COApl^Esr1^ neurons (Extended Fig. 5a-c). Furthermore, we found that mice injected with the hM4Di virus showed a significant increase in anogenital investigation and a significant decrease in the number of active withdrawals (Extended Fig. 5d-f). Since COApl^Esr1^ neurons receive input from the main olfactory bulb^17–19^, we ran two control experiments to ensure that the observed alterations in social behaviour were not due to anosmia. We first ran a hidden food test to assess general olfactory processing^20^. In the test, mice are food restricted overnight and must find a piece of food buried under bedding by using their sense of smell. Inhibition of COApl^Esr1^ neurons did not affect the ability to locate the food (Fig. 3e). Next, we tested the ability of male AGGs to differentiate between males and females^21^. Male mice were placed in an open field arena with a male and female mouse under mesh cups on opposite sides of the arena. We computed a discrimination ratio in which the difference in time spent investigating the male and the female was divided by the total time spent investigation both mice. A negative score indicates more time spent investigating the male and a positive score indicates more time spent investigating the female. We found that inhibiting COApl^Esr1^ neurons did not affect the males’ preference for investigating the female mice (Fig 3f). To further support the idea that COApl^Esr1^ neurons participate in mediating the transition from the appetitive to consummatory phase of aggression, we examined the relationship between the change in attack and investigation duration due to manipulations of COApl^Esr1^ neurons. In mice injected with hM4Di, we found a significant negative correlation between the change in attack duration and the change in investigation duration (Fig 3g). There was a trend in mice injected with hM3Dq for a negative correlation between the increase in attack duration and decrease in investigation duration (Fig. 3h) with no effect observed in mice injected with the mCherry virus (Fig 3i). Lastly, we tested whether inhibition of COApl^Esr1^ neurons alters the motivation to engage in attack behaviour by utilizing the aggression self-administration paradigm^22^. We found that both the mCherry and hM4Di groups learned the task equally well in terms of rewarded trials and the occurrence of attacks (Extended Fig. 5g-i). Following the acquisition of the task, mice were tested using a counterbalanced injection regimen (i.e., CNO/saline). We did not observe any difference in lever presses (Fig. 3j) between the two groups on CNO, suggesting that COApl^Esr1^ cells do not affect the reinforcing properties of social interaction (Fig. 3k). However, we observed a striking decrease in the percentage of rewarded trials which led to an attack (Fig. 3l). We also tested mice on a progressive schedule and did not find a difference in the maximum number of lever presses made for access to an intruder, suggesting that the COApl^Esr1^ cells do not regulate the motivation to work for access to an intruder. Lastly, manipulations of COApl^Esr1^ neurons in female AGGs during the RI test did not affect attack (Extended Fig. 6a-c) or investigation behaviours (Extended Fig. 6d-f). Collectively, these results suggest that the inhibition of COApl^Esr1^ neurons selectively alter the consummatory drive in social interactions in male AGGs.

## Inhibiting COApl^Esr1^ neurons alters network connectivity

**Figure 4:**
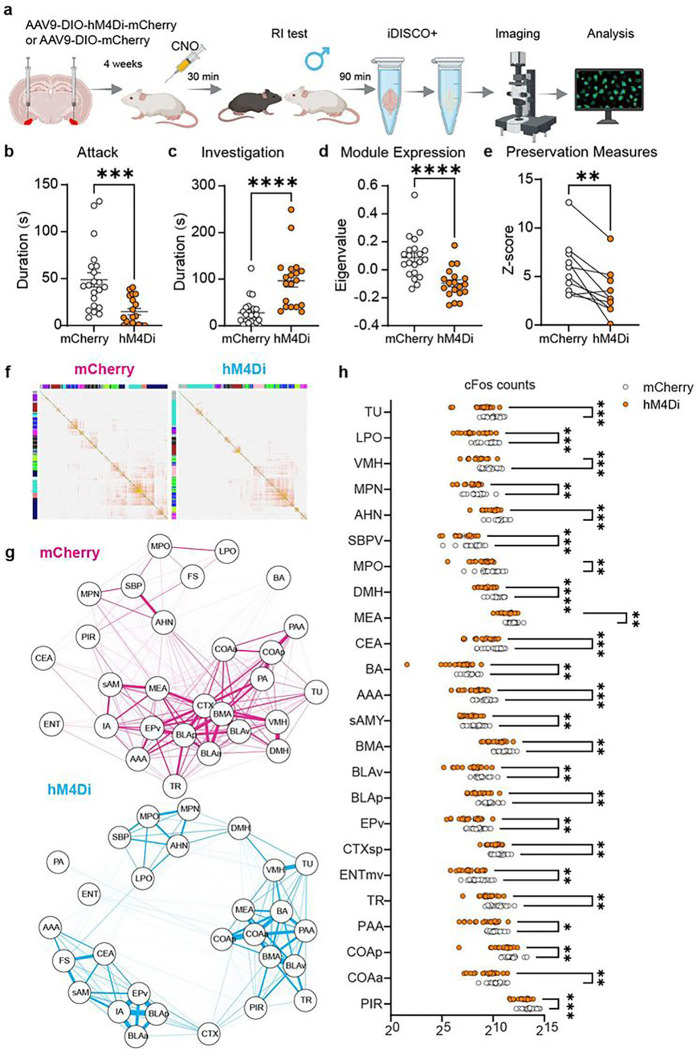
Manipulation of COApl^Esr1^ cells alters connectivity in the pink module. **a**,Experimental timeline. **b-c**, Attack duration (**b**, $t_{\left(38\right)}=3.917$, p=0.0007) and investigation duration (**c**, $t_{\left(38\right)}=4.723$, p<0.0001) change after COApl^Esr1^ cells inhibition. **d-e** mCherry network demonstrated higher pink module expression ($t_{\left(38\right)}=4.442$, p<0.0001) when the two networks were combined, and better preserved the connectivity and density of the pink module than the hM4Di network ($t_{\left(9\right)}=3.983$, p=0.0032) when separately analyzed. Each pair of points connected by a line are a single preservation metric from each network. n=19. (HM4di), 21 (mCherry) **f, g,** Whole brain c-Fos network in mice injected with mCherry (MC) or hM4Di (H4) and network plot of the pink module from the WT AGG network. Regions from the mCherry and hM4Di networks were plotted using the colour label from the original network. **h**, Of the 28 regions in the pink module, inhibition of COApl^Esr1^ cells significant decreased c-Fos expression in 25 (89%) regions. * p < 0.05, ** p < 0.01, *** p < 0.001, **** p < 0.001.

After determining the effect of inhibiting COApl^Esr1^ neurons on aggressive behaviour in males, we sought to investigate how inhibiting this cell population alters the connectivity of the pink module from the AGG network. To this end, we injected 8-week-old male AGGs with AAV-DIO-hM4Di or AAV-DIO-mCherry viruses. Following recovery from surgery, all mice were given an injection of CNO prior to the last RI test and were perfused 90 minutes later (Fig. 4a). As expected, inhibiting COApl^Esr1^ cells decreased attack duration and increased investigation duration (Fig. 4b, c; Extended Fig. 7a-f). Brains were perfused, cleared, imaged, and registered to the Allen brain atlas. We then constructed a network for each group of mice. For visualization, the clusters were organized according to the original AGG network to ensure that the same brain regions would make up similar clusters in the hM4Di and mCherry networks (Fig. 4d–g). As in the modules from Fig. 1, visual inspection indicated a high degree of interconnectedness between amygdalar and hypothalamic subregions under control mCherry conditions. Following inhibition of COApl^Esr1^ cells, we observed high levels of interconnectedness within, but not between, amygdalar and hypothalamic subregions, which is consistent with data from NONs in Fig 1. We also found that inhibiting COApl^Esr1^ cells with hM4Di significantly decreased expression of the pink module relative to the mCherry group (Fig. 4d). We next determined if the hM4Di and mCherry networks differed in how well they preserved the pink module in the original AGG network. To this end, we performed a module preservation analysis separately for each network with the original AGG network serving as the reference network. Using the same metrics from Fig.1, we found that the hM4Di network showed a significant decrease in these metrics compared to the mCherry (Fig. 4e), suggesting that inhibiting COApl^Esr1^ neurons diminished the preservation of the pink module found in the original network. Of the remaining 7 clusters, the hM4Di group only showed a significant decrease in the ‘red’ cluster (Supplementary Fig. 7g). Interestingly, this cluster contains regions which COApl^Esr1^ neurons project to, including the bed nucleus of the stria terminalis (BNST), the anteroventral periventricular nucleus (AVPV), and the substantia innominata (SI). When examining c-Fos counts in individual regions of the pink network, we found that 89% of regions showed a significant reduction in c-Fos cells following inhibition with hM4Di (Fig. 4h and Supplementary Fig. 7h).

## Inhibiting of COApl^Esr1^ neuron outputs alters aggressive social behaviour

**Figure 5:**
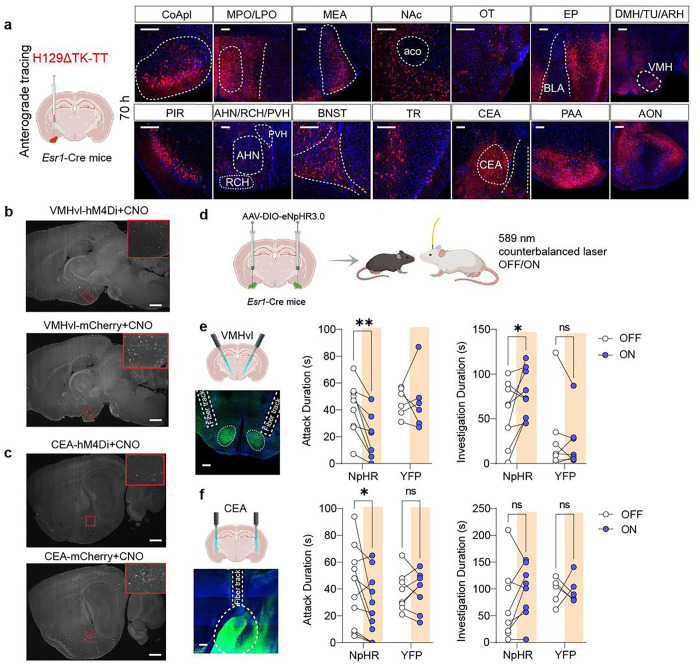
Optogenetic inhibition of regions downstream of COApl^Esr1^ cells alters social behaviour in male AGGs **. a,** Schematic experimental timeline. **b**, Regions which receive monosynaptic input from COApl^Esr1^ cells as revealed by HSV-1 trans-synaptic anterograde tracing. **c-d**, Images of c-Fos^+^ cells in the VMH (**c**) and CEA (**d**) from mice injected with hM4Di or mCherry. **e**, Schematic behavioural setup. **f**, Inhibition of terminals in the VMHvl reduces attack duration in mice injected with NpHR (virus x laser interaction: F(1,14)=7.893, p=0.0139, Sidak’s post-hoc: p=0.0048) and increased investigation duration (virus x laser interaction: F(1,14)=4.920, p=0.0436, Sidak’s post-hoc: p=0.0402). **g**, Inhibition of terminals in the CEA reduces attack duration in mice injected with NpHR (virus × laser interaction: F(1,17)=3.654, p=0.0729, Sidak’s post-hoc: p=0.0239) but has no effect on investigation duration (virus × laser interaction: F(1,17)=0,7045, p=0.4192, Sidak’s post-hoc: p=0.2945) n=9 (VMH NpHR), 7 (VMH YFP). 11 (CEA NpHR), 8 (CEA YFP). * p < 0.05, ** p < 0.01, *** p < 0.001.

We next sought to understand which regions within the pink network downstream of COApl^Esr1^ neurons contributed to the behavioural effects of local manipulation (Fig. 5a). To first identify downstream regions, we injected AAV-DIO-eYFP in COApl^Esr1^ neurons to visualize axonal terminals projecting from COApl^Esr1^ cells. We also injected HSV-1 (H129 TK-TT) to confirm that visualized axonal terminals formed monosynaptic connections with downstream regions of interest (Fig. 5b). Interestingly, many of these regions were previously identified to promote aggressive behaviour, including the ventrolateral portion of the ventromedial hypothalamus (VMHvl), the medial amygdala (MEA), the central amygdala (CEA), and the BNST. Furthermore, we found that this cell population also projected to numerous regions involved in olfaction, such as the piriform cortex (PIR), endopiriform cortex (EP), piriform amygdala area (PAA), post-piriform transition area (TR), and the anterior olfactory nucleus (AON). Interestingly, of the 28 regions in the pink module, COApl^Esr1^ neurons formed monosynaptic connections with 13 of them, indicating that many of these regions are both functionally and physically connected. In addition, of these 13 regions, 12 of them showed a significant decrease in c-Fos following inhibition of COApl^Esr1^ DREADDs (Fig. 4h). Based on this information, we chose to target the VMHvl (Fig. 5c), which is necessary for aggressive behaviour, and the central amygdala (Fig. 5d), which has been shown to modulate jaw movements during biting, a key component of aggressive behaviour. To evaluate whether these circuits alter aggressive behavior, we injected AAV-DIO-NpHR into COApl^Esr1^ neurons of 8-week-old male AGGs and implanted ferrules into either the VMHvl or the CEA (Fig. 5e). Four weeks later, mice were tested for two days in the RI test in which the order of testing (laser on/off) was counterbalanced. We found that inhibiting the COApl-CEA pathway resulted in a significant decrease in total attack duration without affecting social investigation (Fig. 5f, g). In addition, we found that inhibiting the COApl-VMHvl pathway produced a strong reduction in attack duration, and concomitantly increased social investigation, mirroring the effects of locally inhibiting COApl^Esr1^ neurons.

## Discussion

Using an unsupervised approach to analyzing whole-brain c-Fos patterns, we identified the COApl as a key region controlling the transition from social investigation to aggressive behaviour in male, but not in female, mice. Interestingly, many of the regions highly connected to the COApl have previously been identified as part of the “core aggression circuit” such as the MEA, VMH, and the MPOA. Given that these regions are clustered together with many other regions whose function have not been examined in the context of aggression, it is possible that the core aggression circuit is larger than previously considered and requires further investigation. Interestingly, we find that inhibition of the COApl resulted in a significant decrease in c-Fos expressing cells in 79% of the regions within the AGG network (pink module). This is of importance when considering the causal nature of focal manipulations. Understanding the relationship between the manipulated region and other downstream regions is paramount to understanding the relationship between brain-wide activity and behaviour in our studies.^23,24^.

Based on our current understanding of the circuitry mediating aggression, one might have predicted that a loss of function manipulation in the COApl would result in decreased aggression due to the lack of social approach^10,25^. This expectation is perhaps due to previous studies demonstrating that loss of function manipulations in the main olfactory bulb, which projects to the COApl, reduce social investigation and aggression^26,27^. However, our findings that COApl^Esr1^ neurons are more active during RI trials in which an attack occurs, and that this population is specifically active during investigation bouts which precede an attack, suggests that this region is not simply relaying olfactory ‘cues’ to downstream regions. This is further supported by our findings which demonstrate that inhibiting COApl^Esr1^ neurons increasesinvestigation rather than decreasing it without resulting in anosmia. In addition, our findings that COApl^Esr1^ neurons respond to odours from conspecifics, but not from predators, suggests that they are not acting as a simple “threat detector”, which is in line with a previous study demonstrating that the COApl is not involved in fear responses to predator odours^19^. While it was previously reported that the COApl is required for the attraction to non-social odourants such as 2-phenylethanol, this was not cell-type specific^18^. Our data support the idea that COApl^Esr1^ neurons are exclusively activated by odours emitted from conspecifics and therefore shapes the type of social encounter that ensues.

One potential explanation of our findings is that COApl^Esr1^ neurons are involved in the motivation to attack the intruder thereby regulating the rewarding/reinforcing properties of aggression^28^. In support of this explanation, inhibiting COApl^Esr1^ neurons with hM4Di robustly decreased the percentage of rewarded trials which led to an attack in an aggression self-administration task. However, inhibiting COApl^Esr1^ neurons did not change the number of lever presses and mice in both groups worked equally hard for access to a social target, suggesting that their motivation to interact with the intruder—albeit in a less aggressive and more pro-social way—remained intact. Though limited, some previously published studies of other nuclei that make up the pallial amygdala (basolateral, basomedial, cortical and posterior amygdala) support its role in rodent social behavior ^29–31^. For example, inhibition of basolateral amygdala (BLA) inputs to the ventral hippocampus and the medial prefrontal cortex increases affiliative social behaviour in mice, though aggression was not assayed in these studies. Conversely, manipulating the posterior amygdala alters aggressive behaviour without affecting pro-social investigation^32,33^. PAllila amygdala subcircuits have also been implicated in social behavior in human and non-human primate studies. These studies have focused largely on the BLA and lateral amygdala given their extensive connections with the visual system^34,35^, which primates are highly reliant upon during social interactions. Some studies have demonstrated that neurons in the BLA fire when primates make eye contact with one another, and bilateral lesions often reduce fear of others and limits aggression. However, some contradictory results have also been reported, perhaps due to their use of non-specific excitotoxic lesions (reviewed in ^36^). A recent study using the GABA agonist muscimol to reversibly inhibit the BLA, increased affiliative behaviors between pairs of rhesus monkeys, while activating the BLA resulted in social withdrawals without affecting anxiety^37^, mirroring our findings in mice. Furthermore, single unit recordings in humans indicate that neurons in the amygdala are sensitive to the subjective perception of emotions shown in facial stimuli, as opposed to just the features of stimulus^38^. This suggests that the amygdala of different individuals can respond differently to the same sensory cues depending on the behavioural state/belief of the individual. Collectively, these findings have led to the interpretation that the amygdala plays a key role in social perception^39,40^. Thus, it is tempting to speculate that COApl plays a role in the perception of the intruder which is affected by the “aggressive state” of the resident. Internal states have previously been shown to bias perception towards specific actions in other areas of rodent research. For instance, a state of hunger has been shown to enhance the responsivity of the insular cortex and lateral amygdala to sensory cues related to food^41,42^. Additionally, the auditory cortex of mothers is more responsive to pup cries than virgin mice^12,43^. Our research thus provides an important foundation for understanding the neural mechanisms underlying social perception and action selection. Future studies designed to delineate the mechanisms by which internal states and perceptual processes interact to execute distinct behaviours during dyadic social interactions will thus be of high importance.

## Methods

### Animals

Wild-type Swiss-Webster (SW) mice (male and female, 12–15 weeks; Charles River) and *Esr1*-Cre mice (017911, B6N.129S6(Cg)-Esr1^tm1.1(cre)And^/J; Jackson Laboratory) were crossed with SW females to obtain F1 mice used as experimental mice. Intruders for the resident-intruder (RI) test were 8–12-week-old male or female C57/BL6J mice (Jackson Laboratory). All delivered mice were allowed 1 week of acclimation to the housing facility prior to any experimental protocol. At 8 weeks, mice were separated from their littermates and paired with a member of the opposite sex for sexual experience. Females were paired with castrated SW males (8–12 weeks; Charles River) to prevent pregnancy. All mice were maintained on a 12/12 h light/dark cycle (07:00–19:00) with ad *libitum* access to food and water. Housing and experimental rooms were maintained at 20–22 °C and 40–60% humidity. Experiments were conducted during the light phase. Procedures were performed in accordance with the National Institutes of Health Guide for Care and approved by the Use of Laboratory Animals and the Icahn School of Medicine at Mount Sinai Institutional Animal Care and Use Committee.

### RI Test

Mice were screened using protocols adapted from previous studies^3,44^. Briefly, cage tops were removed and replaced with Plexiglas covers to monitor trials. A novel C57BL/6J mouse matching the sex of the resident was introduced into each cage and mice were allowed to freely interact for 5 min. After 5 min elapsed, intruder mice were returned to their home cages and, in the case of female resident-intruder trials, cohabiting male mice were returned to their home cages. All videos were recorded for later analysis. Resident behaviors were manually annotated using Observer XT 11.5 (Noldus Interactive Technologies).

### Aggression Self-Administration (SA) Test

Aggression SA testing was conducted as previously described^45^. Briefly, SW resident mice were placed in standard Med Associates operant chambers and underwent 8 trials per day for 16 days in which they could press a lever (FR1) in order to receive access to a subordinate same-sex mouse through a guillotine door into their operant chamber. A house light illuminated the chambers during trials, and an inactive lever was extended at all times. At the beginning of a trial, the house light turned on, and a is lever extended 10 s later. If the resident pressed for a reward, a 2 s tone played and a guillotine door next to the active lever opened for 12 s. A same-sex C57BL/6J intruder manually pushed into the chamber, and the intruder was removed at the end of the trial. If residents did not press, the active lever retracted after 60 s. All chambers also had recessed food pellet receptacles with beam-break registered entries ports. We conducted all Progressive Ratio (PR) tests using the same parameters we used for self-administration training, except for the trial design. Following reward obtention (number of required active lever presses for a given trial), the automatic guillotine door was opened for 10 s and the same-sex intruder (C57BL/6J) was manually pushed inside the main operant chamber. Immediately after the automatic door closed, the active/inactive levers were re-extended. The intruder mouse was removed from the chamber following an attack or after 30 s had elapsed. The PR session was terminated if no reinforced responses occurred after a total duration of 20 min had elapsed. Throughout the sessions, for each trial, the number of responses required to activate the guillotine door and gain access to the intruder was incremented following an exponential progression (R=5×e0.12*P-5, where P is the previous ratio). The breakpoint value corresponds to the total number of rewards (number access to the intruder during the whole session). *Esr1*-cre mice transfected in the COApl using AAV9-DIO-hM4Di-mCherry or AAV9-DIO-mCherry (see titer, surgeries information and coordinates below) were administered either saline (SAL) or Clozapine-N-Oxide (CNO), counterbalanced over the two days of the PR.

### Hidden Food Test

Mice were habituated to and allowed to consume a palatable food (i.e., Reese’s mini peanut butter cup) two days prior to being tested. Mice were then food-restricted the night before the test. Mice were tested under red light and placed in a clean mouse cage. The food was placed 8 cm deep in the bedding of the cage and the time to find the food was recorded with a stop-watch.

### Sex Discrimination Test

Male and female C57/BL6J mice were used as target mice and placed on opposite sides of an open field enclosure in mesh cups. Test mice were placed in the open field and allowed to explore the arena for five minutes under red light. Mice were recorded with an overhead Ethovision camera and time spent interacting with each mouse was scored offline manually. To calculate the discrimination ratio, the total time spent interacting with both mice was divided by the difference between time spent with the female and male. A positive discrimination ratio indicates more time spent with the female, a negative ratio indicates more time spent with the male.

### Perfusion and brain tissue processing

For iDISCO+, mice were injected with 10% chloral hydrate and perfused transcardially with ice-cold 1× PBS (pH 7.4), followed by fixation with cold 4% paraformaldehyde in 1× PBS. Brains were post-fixed for 12 h in the same fixative at 4 °C. For FISH, brains were rapidly removed and flash-frozen in −30 °C isopentane for 5–10 s then kept at −80 °C until sectioning. Slices were sectioned at 15 μm using a cryostat (Leica). Animals injected with H129ΔTK-TT were perfused 48 h after injection.

### *In Situ* Hybridization and Imaging

For FISH, RNAscope Multiplex Fluorescent Kits (Advanced Cell Diagnostics) were used according to the manufacturer’s instructions. Fresh-frozen brains were mounted on slides at 15 μm thickness, fixed for 15 min in cold 4% PFA and dehydrated serially with 50, 70 and 100% EtOH/H_2_O for 2 min each, followed by 20 min Protease IV (RNAscope) treatment. Proprietary probes (Advanced Cell Diagnostics) for Fos (316921, accession no. NM_010234.2), *Vglut1* (SLC17A7-C3, accession no. NM_182993.2) and *Esr1* (478201-C2, accession no. NM_007956.5 ) were hybridized at 40 °C for 2 h then subjected to a series of amplification steps at 40 °C (1-FL, 30 min; 2-FL, 15 min; 3-FL, 30 min; 4-FL, 15 min). Reagent Alt-A was used for the fourth amplification step, with Channel 1 at 488 nm, Channel 2 at 550 nm and Channel 3 at 647 nm. Finally, slides were treated for 30 s with DAPI and immediately coverslipped with Eco-Mount. All slices were imaged using a Zeiss LSM 780 confocal microscope. Cells from all ISH images were counted blindly across groups. Cells with at least 5 puncta for each probe were considered to be positive for the probe of interest.

### iDISCO+ staining, imaging and ClearMap analysis

The iDISCO+ staining protocol was modified from http://www.idisco.info. Fixed whole brains were incubated with the primary Fos antibody (no. 226003, 1:1,000, Synaptic Systems) and secondary donkey anti-rabbit IgG (H+L) Highly Cross-Adsorbed Secondary Antibody, Alexa Fluor 647 (no. A-31573, 1:1,000, Thermo Fisher Scientific) for 7 days each. A LaVision lightsheet microscope with zoom body was used for half-brain sagittal scanning, with dynamic focus and a step size of 4 μm. Cleared brains were processed as previously described using ClearMap^13^. Fos^+^ cells were quantified using the cell detection module, with cell detection parameters optimized and validated based on the intensity and shape parameters of the signal. The autofluorescence channel was aligned to the Allen Institute’s Common Coordinate Framework using the Elastix toolbox. To compare cell counts between AGG and NON animals in both sexes and hM4Di vs. mCherry, a negative binomial regression was applied using the glm.nb function from the MASS package in R. Group classifications were dummy coded (0 for the AGG group and 1 for the NON group). The maximum-likelihood coefficients α and β were determined through iterative reweighted least squares. A significant β means that group status is related to cell count number at the specified region of interest. Z-scores correspond to this β coefficient, normalized by its sample standard deviation. P values were corrected for multiple comparisons using the Benjamini–Hochberg procedure to decrease false discovery rate. Q-values below 0.05 were considered significant.

### Stereotaxic surgery and viral gene transfer

*Esr1*-Cre mice (8–10 weeks old) were anaesthetized with an intraperitoneal injection of ketamine HCl (100 mg kg^−1^) and xylazine (10 mg kg^−1^) and positioned on a stereotaxic instrument (David Kopf Instruments). In the lateral portion of the COAp (COApl) (bregma: AP −1.7 mm; ML ±2.8 mm; DV −5.9 mm), 0.3 μL of virus was bilaterally infused using 33-gauge Hamilton needles over 5 min, with needles left in place for 5 min after injection. For virus delivery, 0.3 μl of AAV8-hSyn-DIO-hM3D-mCherry (2.0 × 10^12^ vg mL^−1^, no. 44361-AAV8, Addgene), AAV9-hSyn-DIO-hM4D-mCherry (2.0 × 10^12^ vg mL^−1^, no. 44362-AAV9, Addgene) or AAV9-hSyn-DIO-mCherry (2.0 × 10^12^ vg mL^−1^, no. 50459-AAV9, Addgene) was injected into the COApl. We also injected 0.3 μL AAV9-hSyn-DIO-hM4D-mCherry (2.0 × 10^12^ vg mL^−1^, no. 44362-AAV9, Addgene) into the COApm (bregma: AP −2.4 mm; ML ±2.0 mm; DV −5.6 mm) For anterograde tracing, 0.3 μL of AAV9-hSyn-DIO-EYFP (2.0 × 10^12^ vg mL^−1^, no. 50457-AAV9, Addgene) or 0.2 μl of H129ΔTK-TT (4.0 × 10^9^ vg mL^−1^, Center for Neuroanatomy with Neurotropic Viruses) was injected unilaterally into the COApl. For optogenetic manipulations, AAV9-Ef1a-DIO eNpHR3.0-EYFP (3.0 × 10^12^ vg mL^−1^, no. 26966-AAV9, Addgene) or AAV9-EF1a-DIO-EYFP (3.0 × 10^12^ vg mL^−1^, no. 20298-AAV9, Addgene) was injected into the COApl. All AAV injections were performed 3–4 weeks before behavioural experiments. For optogenetic (ChR2) and FP experiments, cannulae (ChR2: MFC_200/240–0.22_3mm_MF1.25_FLT; FP: MFC_200/250–0.57_3mm_MF1.25_FLT) were implanted at the same time as viral delivery (for COApl local, fibres were implanted 0.2 mm above the injection site). For optogenetic (eNpHR3.0) experiments of COApl terminal stimulation, cannulae (MFC_200/240–0.22_MF1.25_FLT, 6 mm for VMHvl, 5 mm for CEA) were bilaterally implanted into the VMHvl (from bregma: AP −1.7 mm; ML ±2.5 mm; DV −5.5 mm, 20° angle), or CEA (from bregma: AP −1.5 mm; ML ±2.5 mm; DV −4 mm, 0° angle). For secure fixture of the optic fibre, dental cement (Grip cement; Dentsply) was added to the skull and around the fibres.

### DREADD manipulation

CNO (1 mg kg^−1^, Tocris) was given intraperitoneally 30 min before the RI test, open field, aggression self-administration, hidden food and sex discrimination tests.

### Optogenetics manipulation

For yellow (eNpHR3.0) light stimulation, optical fibres (BFP(2)_200/220/900–0.22_4m_FCM-2xMF1.25, Doric Lenses) were connected to a 589 nm yellow laser diode (no. MGL-III-589–50mW, Opto Engine LLC) using a patch cord with a FC/PC adaptor (no. MFP_200/240/900–0.22_4m_FC-MF1.25, Doric Lenses). A function generator (no. 33220A, Agilent Technologies) was used to generate constant orange light was used for eNpHR3.0 experiments during the 5 min RI test. For all optogenetics tests, experimental mice were habituated to patch cords for 2 days before testing in RI. For RI experiments, mice were tested over 2 days, counterbalanced under laser-on and -off conditions.

### Fibre photometry

Fibre photometry was performed according to the Neurophotometrics manual and published protocols^5,44^. A fibre-optic patch cord was attached to the implanted cannula with cubic zirconia sleeves covered with black tubing. The opposite end of the cable was coupled to a Neurophotometrics LED port. The open-source Bonsai programme was used to control the system; 470 and 415 nm LED lights were used for GCaMP6s signal and autofluorescence measurement. Light at the fibre tip ranged from 40 to 80 μW and was constant across trials over testing days. Simultaneous recording of 40 fps from both 470 and 415 nm channels was achieved phase to phase and visualized via Bonsai. Three weeks after virus injection and ferrule implantation, mice were tested in the RI test and were presented different odours in a counterbalanced manner. Once connected to the apparatus, mice were allowed to rest and habituate for 3–5 min before starting. For the RI test and odour presentation, we recorded Ca^2+^ fluorescence during 2 min of baseline activity without an intruder, followed by 5 min of intruder or odor exposure. The 415 nm channel served as the control channel and was subtracted from the GCaMP6s channel to eliminate autofluorescence, bleaching and motion effects. Change in fluorescence (ΔF/F) was computed by subtracting the average value during the final minute of baseline recording and then dividing the resulting value by the averaged value during the final minute of baseline. This value was then z-scored by subtracting the average ΔF/F from each value and dividing by the standard deviation. Behavioural data were manually annotated in Ethovision and time stamps were aligned with fluorescence recording.

### Weighted correlation network analysis

For network construction, we followed previously published guidelines^14^. Briefly, a n×n adjacency matrix was created which encodes the connection strengths between pairs of nodes (brain regions), using the power adjacency function: $A_{ij}=\left|s_{ij}\right|{\;}^{\beta }.\;A_{ij}$ refers the adjacency matrix and $s_{ij}$ refers to the correlation between regions i and j raised to the power of β. The correlation is raised to the power of β in order to reduce the influence of potentially specious correlations. To measure the similarity between the nodes for clustering the topological overlap measure (TOM) was used: $\omega_{i,j}=\frac{l_{ij}\;+\;a_{ij}}{min\left\{k_{i,}k_{i}\right\}+1-a_{ij}}.\omega_{i,j}$ refers to the TOM matrix, $l_{ij}=\sum_{u}\;a_{iu}a_{uj}$ and $a_{ij}={\left|s_{ij}\right|}^{\beta }$. In words, this is the sum of the product of the shared connections between regions i and j plus the direct connection between regions i and j. This value is divided by the denominator to achieve a value between 0 and 1. In order to compare the expression of these modules between sexes within a network, a singular value decomposition (SVD) was computed on each module. The SVD is defined as: $X^{n}=U\;*\;S\;*\;V^{T}\cdot X^{n}$ is the TOM matrix for the n^th^ module in the network, U and $V^{⊤}$ are matrices of orthonormal vectors, and S is a diagonal matrix of eigenvalues that denote how much variation is accounted for by each column of U and V. To determine which modules differed between AGG and NON networks, or whether the mCherry and hM4Di networks conserved module connectivity and density, we used the module preservation function. For comparisons between networks we focused on the following measures for connectivity: Z.cor KIM, Z.cor.KME, Z.cor.KMEall, Z.cor.cor, and Z.cor.MAR. For density measures, we focused on Z.propVarExplained, Z.meanSignAwareCorDat, Z.meanAdj and Zmean.MAR. All Z values were derived by randomly permuting the module labels in the test network and calculating the corresponding preservation metric. The average value of each statistic from the permutations is subtracted from the observed statistic and divided by the standard deviation of the statistic from the permutations. For a detailed explanation of the preservation measures, see table 2. All of the above steps were performed using the WGCNA package in R.

### Statistical analysis

All statistical tests and associated information are reported in the Figure legends. All t-tests and two-way ANOVAs were performed using GraphPad Prism software (GraphPad Software Inc.). Two-way repeated-measures ANOVA analysis was followed by Šídák’s multiple-comparisons test for post-hoc analysis. For comparisons between groups for region by region iDISCO+ analysis, P values were corrected for multiple comparisons using the Benjamini–Hochberg procedure to reduce false discovery rate. Q values below 0.05 were considered significant.
